# Supplementary material for: A Three-Year Longitudinal Study Comparing Bone Mass, Density, and Geometry Measured by DXA, pQCT, and Bone Turnover Markers in Children with PKU Taking L-Amino Acid or Glycomacropeptide Protein Substitutes
Source: Nutrients. 2021 Jun 17;13(6):2075. doi: 10.3390/nu13062075 (PMC8233747; doi:10.3390/nu13062075)
Supplement: Supplementary file 1 [file nutrients-13-02075-s001.zip › nutrients-1261066-supplementary.pdf]

Supplementary Table S1. Differentiation of median DXA z scores (range): lumbar vertebrae L2-L4 areal bone mineral density (L2-L4 aBMD); lumbar vertebrae L2-L4 bone mineral apparent density (L2-L4 BMAD) and total body less head areal bone mineral density (TBLH BMDa) by gender. Median value (range) for total body bone mineral content (BMC) by gender.

		Female		Male	
Z score		Enrolment	36 months	Enrolment	36 months
L2-L4 BMDa g/cm^2	L-AA	0.05 (-0.5, 0.2)	-0.4 (-0.6, -0.1)	-0.2 (-0.9, 0.35)	-0.7 (-1.2, 0.1)
	GMP100	0.3 (-0.5, 0.9)	0.1 (-0.7, 1.0)	-0.7 (-0.9, 0.1)	-0.9 (-0.9, 0.4)
	GMP50	0.1 (-0.6, 0.4)	0.1 (-0.4, 0.4)	-0.2 (-0.4, 0.1)	-0.2 (-0.7, -0.1)
L2-L4 BMAD g/cm^3	L-AA	-0.05 (-0.4, 0.5)	-0.5 (-0.8, -0.1)	-0.4 (-0.8, 0.3)	-0.9 (-1.4, -0.1)
	GMP100	0.4 (-0.1, 0.7)	0 (-0.4, 0.6)	-0.4 (-1, 0.2)	0.2 (-0.5, 0.4)
	GMP50	0.15 (-0.7, 1.3)	-0.3 (-0.5, 0.4)	-0.2 (-0.5, -0.1)	-0.2 (-0.2, -0.1)
TBLH BMDa g/cm^2	L-AA	0 (-0.3, 0.1)	-0.2 (-0.3, 0.)	-0.3 (-0.6, -0.1)	-0.2 (-0.4, -0.1)
	GMP100	-1 (-1.3, -0.7)	-0.4 (-0.7, -0.2)	-0.6 (-0.7, -0.3)	-0.4 (-0.7, -0.3)
	GMP50	-1.1 (-1.6, -0.6)	-0.7 (-0.9, -0.5)	-0.7 (-0.9, -0.1)	-0.6 (-0.9, -0.1)
**Median values (range) for Total BMC g**					
		Enrolment	36 months	Enrolment	36 months
Total body BMC g	L-AA	1266 (608, 1610)	1690 (990, 1779)	984 (761, 1454)	1472 (1192, 1817)
	GMP100	759 (666, 847)	1287 (1103, 1476)	887 (704, 1210)	1405 (1167, 1871)
	GMP50	578 (528, 646)	1009 (956, 1113)	675 (567, 733)	1023 (1010, 1186)

Legend: L2-L4 BMDa; L2-L4 lumbar spine vertebrae areal bone mineral density; L2-L4 BMAD; L2-L4 lumbar spine vertebrae bone mineral apparent density; TBLH, BMDa total body less head areal bone mineral density; BMC total body bone mineral content, DXA, dual-energy x-ray absorptiometry; L-AA, amino acids; CGMP, casein glycomacropeptide; CGMP100, children taking all their protein substitute as casein glycomacropeptide; CGMP50, children taking a combination of casein glycomacropeptide and amino acids.

Statistical significance (p value <0.05) was not reported for any parameter

Supplementary Table S2. Differentiation of peripheral quantitative computerised tomography(pQCT) z scores (range) by gender at 36 months

**Z scores**	**Treatment**	**Female z score (range)**	**Male z score (range)**
Trabecular density 4%	L-AA	-0.4<br>(-0.9, 0)	-0.6<br>(-1.4, -0.1)
	GMP100	-1.0<br>(-1.2, 0.9)	-0.6<br>(-1.5, -0.2)
	GMP50	-1.0<br>(-1.4, 0.9)	-1.0<br>(-1.2, -0.3)
Total.density 4%	L-AA	-0.1<br>(-0.5, 0.8)	-0.7<br>(-0.9, 0.3)
	GMP100	-1.1<br>(-1.2, -0.9)	-0.7<br>(-0.8, -0.3)
	GMP50	-0.8<br>(-1, -0.6)	-0.5<br>(-0.8, 0.2)
SSI<br>(Strength, strain index)	L-AA	0.5<br>(-0.2, 1.3)	0.2<br>(-0.4, 0.6)
	GMP100	-1.0<br>(-1.3, -0.3)	0.1<br>(-1.1, 1)
	GMP50	0<br>(-0.2, 0.3)	-0.7<br>(-1.3, 1.2)
Cortical density 66%	L-AA	-0.4<br>(-1.1, 0.3)	-0.2<br>(-0.9, 0.5)
	GMP100	0.2<br>(0.1, 0.2)	0.1<br>(-0.3, 0.7)
	GMP50	-0.4<br>(-1.4, 0.1)	-0.5<br>(-0.7, -0.1)
Muscle area 66%	L-AA	-1.7<br>(-2.2, -0.8)	-0.8<br>(-1.3, -0.5)
	GMP100	-1.7<br>(-1.8, -1.6)	-0.7<br>(-0.8, -0.1)
	GMP50	-1.2<br>(-1.4, -0.6)	-0.9<br>(-1.4, -0.5)
Bone area 66%	L-AA	2.5<br>(1.7, 2.8)	1.8<br>(1.5, 3.9)
	GMP100	1.6<br>(1.4, 2.3)	3.7<br>(1.4, 4.2)
	GMP50	1.5<br>(0.9, 2)	0.5<br>(0.1, 1.3)
Fat area 66 %	L-AA	1.3<br>(0.7, 2.1)	0.5<br>(0.1, 2.4)
	GMP100	0.9<br>(0.7, 1.0)	-0.2<br>(-0.7, 0.7)
	GMP50	1.6<br>(0.7, 1.8)	0.5<br>(-0.2, 1.0)
Bone area/muscle area 66%	L-AA	1.2<br>(0.5, 1.9)	0.2<br>(0.2, 1.1)

	GMP100	0.5<br>(0.2, 1.0)	0.3<br>(-0.3, 1.1)
	GMP50	0.4<br>(-0.9, 0.6)	-0.5<br>(-1.4, -0.1)

Legend: L-AA, amino acids; CGMP, casein glycomacropeptide; CGMP100, children taking all their protein substitute as casein glycomacropeptide; CGMP50, children taking a combination of casein glycomacropeptide and amino acids; pQCT, peripheral quantitative computerised tomography.

Statistical significance (p value <0.05) was not reported for any parameter
